# Recurrent Ameloblastoma Involving Fibula Neo-mandible: Management with Digital Planning and Reconstruction Using a Contralateral Free Fibula Flap

**DOI:** 10.7759/cureus.7880

**Published:** 2020-04-29

**Authors:** Suzanne M Beecher, Paul Lennon, Michael O'Shaughnessy, Conor P Barry

**Affiliations:** 1 Plastic & Reconstructive Surgery, Cork University Hospital, Cork, IRL; 2 Ear, Nose & Throat Surgery, St. James’s Hospital, Dublin, IRL; 3 Maxillofacial Surgery, St. James's Hospital, Dublin, IRL

**Keywords:** ameloblastoma, free fibula flap, recurrence, mandible, digital planning, reconstruction

## Abstract

Ameloblastoma is a locally aggressive tumor that most commonly arises in the mandible. It has a high rate of recurrence if inadequately excised. We report a case of a patient who developed recurrence of his ameloblastoma in his fibula flap mandibular reconstruction despite clear resection margins 23 years after resection. This is the first reported case of recurrent ameloblastoma in a neo-mandible reconstruction in the setting of negative margins. We discuss its surgical management using digital planning and reconstruction using a contralateral free fibula flap. Ameloblastoma is a locally aggressive entity that requires complete excision. Recurrence can even occur in the reconstruction, which can present a challenge to manage. Consideration should be given to repeat excision and second osseous flap reconstruction.

## Introduction

Ameloblastomas are the most common odontogenic tumor of the jaws, with 80% of tumors occurring in the mandible [1]. It is a benign entity that is usually painless. However, it can be locally aggressive and lead to significant dysfunction, including displacement of teeth, malocclusion, pathological fracture, and facial asymmetry [2].

While conservative surgical management of ameloblastomas (including marsupialisation, curettage and enucleation, with or without peripheral ostectomy) is associated with very high recurrence rates, the recurrence rate associated with en bloc resection is rare [3-5]. To minimize the risk of recurrence, surgical resection should include a clinical margin of 1-2 cm [6]. Recurrence rates after inadequate surgical excision may be as high as 90% [7]. 

There are sporadic reports in the international literature of recurrence despite en bloc resection but the status of the resection margins is normally unclear. We report a case of a patient who developed recurrence of his ameloblastoma in his fibula flap mandibular reconstruction despite clear resection margins and discuss its reconstruction.

## Case presentation

**Figure 1:**
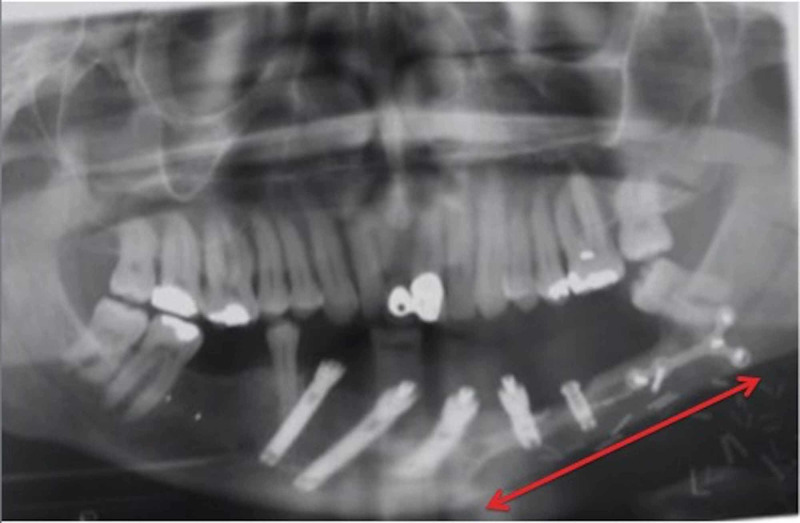
After initial segmental resection and free fibula flap reconstruction (arrow).

A 59-year-old man was referred to the Head and Neck Unit with swelling of the mucosa of his left mandible and exfoliation of his dental implants. He had a history of previous segmental resection of the left mandible and free fibula flap reconstruction from the right lower leg for an ameloblastoma 23 years previously. Histological examination confirmed complete excision with negative surgical margins. The patient subsequently proceeded to implant insertion for dental rehabilitation (Figure 1).

**Figure 2:**
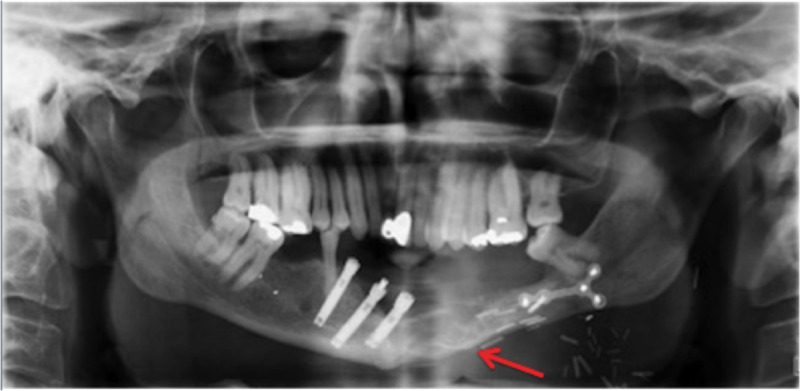
At presentation (2018) with recurrent ameloblastoma (arrow) involving the previous free fibula flap.

At re-presentation, he was investigated with plain film X-ray and CT imaging of his mandible (Figure 2).

The imaging indicated recurrence within his fibula flap neo-mandible. The mandibular mass was biopsied and histology confirmed tumor recurrence. After digital surgical planning, the patient proceeded to radical resection of the recurrent tumor with wide resection margins, but sparing the right mental nerve to preserve sensation of the contralateral lip and chin.

**Figure 3:**
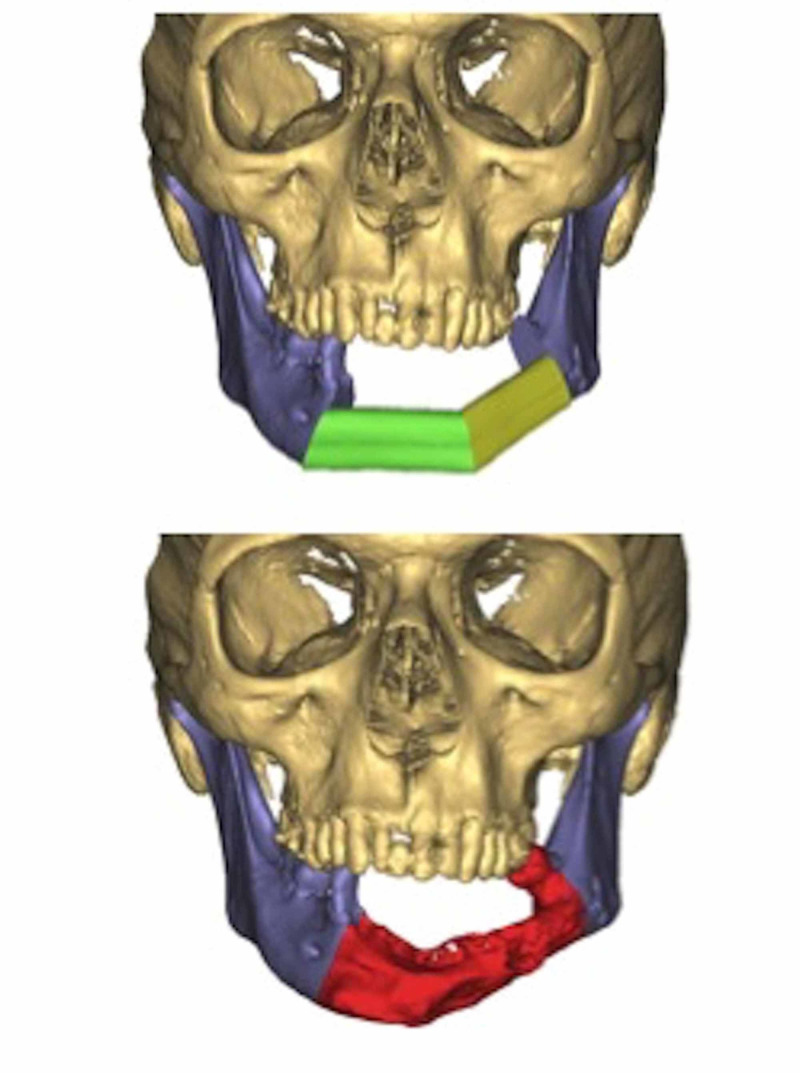
Digital planning of the planned resection.

**Figure 4:**
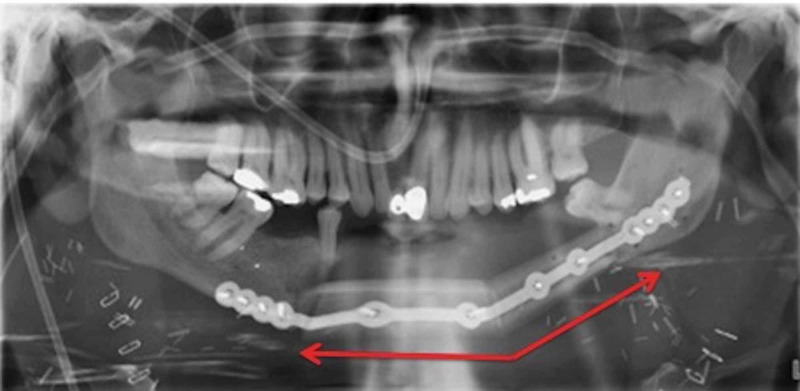
After repeat segmental resection and second free fibula flap reconstruction with osteotomy (arrow).

Figure 3 illustrates the digital planning of the case. Successful microvascular bony reconstruction of the resulting Brown Class 3 defect with restoration of facial contour was achieved using the contralateral fibula (Figure 4).

## Discussion

En bloc resection of ameloblastomas is the gold standard, particularly where there has been failure of more conservative surgical approaches. Because they can reach a large size before becoming symptomatic, en bloc resection normally involves segmental resection of the mandible. While this is associated with significant morbidity, patients can normally be reassured that recurrence of their tumor is exceedingly rare. There are sporadic reports in the international literature of recurrence despite segmental resection [5]. Ameloblastomas most commonly recur within five years, with Almeida et al. suggesting that patients should undergo X-ray screening every six months for five years, then yearly for a further five years [4]. Recurrence after a significant time period is rare, but case reports exist in the literature. Belli et al. reported a case of recurrence after almost 50 years from initial resection [1].

Recurrence can occur in the native remaining mandible, as well as in the mode of reconstruction. Eckardt et al. reported cases of recurrence in nonvascularized bone grafts; however, they acknowledged that adequacy of the surgical margins on histological analysis could not be determined [8].

Basat et al. first described ameloblastoma recurrence in a free fibula flap reconstruction [9]. This occurred seven years after segmental resection and reconstruction. No comment was made on the adequacy of the segmental resection margins. The patient underwent excision of the disease, but opted against reconstruction.

Sharma et al. also described recurrence in a free fibula flap reconstruction [10]. No comment again was made on the adequacy of the surgical margins. In this case report, the patient represented early within six months postoperatively with hardware loosening and exposure. Recurrence was detected at this time. There was no comment on the subsequent management of the recurrence.

Various methods for reconstruction of the resulting defect have been reported, including titanium plates and nonvascularized bone grafts. However, where microvascular expertise is available, bony free tissue transfer is normally advocated, especially for defects over 6 cm. In this case, vascularized bone was essential as an osteotomy was required to form the anterior neo-mandible and a soft tissue component was required to reconstruct the resected mucosa. 

The posterior mandible (Brown Class 1) is the most commonly affected site for the development of an ameloblastoma. In these cases, we favor the deep circumflex iliac artery (DCIA) flap [11]. The volume and natural curvature of the DCIA flap can perfectly restore the contours of the body of the mandible and allow for dental implant rehabilitation. 

 The free vascularized scapula flap can also been used in mandibular reconstruction [12]. For longer defects, especially those involving the anterior mandible, as in this case, where an osteotomy may be required, we have found the fibula flap to be preferable for its length of both bone segment and pedicle. The free fibula flap is a common reliable method of mandibular reconstruction. It can be osteotimized to achieve a good cosmetic and functional outcome. Osseointegrated implants can also be placed successfully at the time of reconstruction [13].

## Conclusions

To date, there are only two cases in the literature reporting recurrent ameloblastoma in the free fibula flap after segmental resection and reconstruction. Neither of these cases reported on the adequacy of the initial surgical resection. We present the first described case of recurrent ameloblastoma involving a fibula neo-mandible in the presence of adequate initial surgical margins. We also present the first case of management of reconstruction in the setting of recurrence in a fibula neo-mandible and demonstrate that it is possible to achieve a good outcome using a contralateral fibula flap. The case illustrates the value of digital planning in reconstruction of bony defects of the jaws resulting in successful reconstruction using the contralateral fibula.
